# Pharmacy-led interventions to reverse and prevent prescribing cascades in primary care: a proof-of-concept study

**DOI:** 10.1007/s11096-025-01873-8

**Published:** 2025-02-15

**Authors:** Atiya K. Mohammad, Jacqueline G. Hugtenburg, Yildiz Ceylan, Marcel Kooij, Saskia Knies, Patricia M. L. A. van den Bemt, Petra Denig, Fatma Karapinar-Carkıt

**Affiliations:** 1 Department of Clinical Pharmacy, OLVG Hospital, Amsterdam, The Netherlands; 2 Department of Clinical Pharmacy and Pharmacology, University Medical Centre Groningen, Groningen, The Netherlands; 3 Department of Clinical Pharmacology and Pharmacy, Amsterdam UMC, Location VUMC, Amsterdam, The Netherlands; 4 Community Pharmacy, Benu Apotheek Wester, Amsterdam, The Netherlands; 5 Community Pharmacy, Service Apotheek Koning, Amsterdam, The Netherlands; 6 The National Health Care Institute (Zorginstituut Nederland), Diemen, The Netherlands; 7 Department of Clinical Pharmacy and Toxicology, Maastricht University Medical Center+, Maastricht, The Netherlands; 8 Department of Clinical Pharmacy, CARIM, Cardiovascular Research Institute Maastricht, Maastricht University, Maastricht, The Netherlands

**Keywords:** Adverse drug reactions, Community pharmacy service, Pharmacotherapy, Pharmacy research, Prescribing cascades

## Abstract

**Background:**

Prescribing cascades occur in clinical practice when a medication causes an adverse drug reaction (ADR), which is addressed by prescribing additional medication.

**Aim:**

The aim was to provide proof-of-concept for pharmacy-led interventions to reverse or prevent prescribing cascades.

**Method:**

Two community pharmacies each tested two approaches. To reverse prescribing cascades, ten cascades were selected from literature. Dispensing records were screened to identify patients with these cascades. To prevent prescribing cascades, patients who started medications associated with five of these cascades were telephoned one month after their first dispensing to discuss ADRs. Pharmacists assessed the need to intervene together with prescribers. Primary outcome was the proportion of patients with a treatment change initiated. Secondary outcomes were time investment, potential cost-savings, and pharmacists’ experiences.

**Results:**

To reverse prescribing cascades, 24 patients were included. For eight the prescriber was consulted, resulting in the reversal of three cascades. Forty-four patients were included to prevent prescribing cascades. Six of them experienced an ADR that could lead to a prescribing cascade. For two patients interventions were conducted to prevent this. The estimated time investment to identify patients possibly in need of intervention was 4.5 h for the reversing approach and 4.8 h for the preventing approach, while follow-up actions required 1.8 h and 0.5 h, respectively. Both approaches could be cost-saving. Pharmacists considered both approaches relevant but identified a knowledge gap on how to intervene for some cascades.

**Conclusion:**

Pharmacy-led interventions may reverse and prevent prescribing cascades, but more efficient screening methods and tools are needed before further implementation.

**Supplementary Information:**

The online version contains supplementary material available at 10.1007/s11096-025-01873-8.

## Impact statements

- Reversing and preventing prescribing cascades can be initiated by pharmacists, which has the potential to lead to less inappropriate prescribing of medication.
- The time investment to conduct interventions should be shortened, particularly by reducing the time needed to screen for patients at risk of a potential prescribing cascade, e.g., through automated procedures, including questionnaires for patients.
- Pharmacists may need more knowledge and information to reverse and prevent prescribing cascades.

## Introduction

A prescribing cascade occurs when medication causes an adverse drug reaction (ADR), which is subsequently addressed by prescribing additional medication [1, 2]. Many prescribing cascades are problematic, causing more harm than benefit to the patient's health [3]. Both the burden for the patient and the healthcare system can increase, as prescribing cascades may lead to additional doctor visits, medication costs, and extra pharmacy services [2, 4]. A recent review identified 79 prescribing cascades with strong and moderate evidence of occurrence in ambulatory care [5]. Also in the Netherlands, evidence was found for 41 potentially problematic prescribing cascades in primary care [6]. Therefore, it is important to develop approaches to reverse existing prescribing cascades and prevent the prescribing of unnecessary medication for ADRs.

A scoping review described possible strategies to reverse and prevent prescribing cascades, including tools for increasing awareness and detecting prescribing cascades, and step-wise protocols to detect and resolve prescribing cascades [7]. For example, the ThinkCascades tool includes a list of nine clinically relevant prescribing cascades, which can raise awareness [8]. Piggott and colleagues described clinical process mapping as a strategy to detect and prevent prescribing cascades [8, 9]. Once detected, a prescribing cascade can be reversed by adjusting the initial medication causing the ADR. Adjustments may involve reducing the dose, discontinuing the initial medication or switching to other medication [10]. To prevent prescribing cascades, timely detection of related ADRs is needed. Clinical decision support tools and medication reviews can be successful in decreasing potentially inappropriate medication and sometimes ADRs [11, 12]. Also, various types of medication reviews conducted by pharmacists and pharmacist-led services can decrease drug-related problems and lower ADR rates [13–17]. However, a recent survey among community pharmacists identified the need to provide resources to help identify and manage prescribing cascades [18]. So far, there are no studies that have tested the potential of pharmacy-led strategies focusing on reducing prescribing cascades. It thus remains unclear whether pharmacists can reverse or prevent prescribing cascades when offered support to do so.

### Aim

This study aimed to provide proof-of-concept for implementing two pharmacy-led approaches in primary care, i.e., one to reverse and one to prevent prescribing cascades. Specific aims were to (a) demonstrate the pharmacist’s ability to initiate interventions to reverse or prevent prescribing cascades after receiving resources to support this, (b) estimate the associated time investment and potential cost-savings, and (c) describe the experiences of pharmacists with implementing these approaches.

### Ethics approval

The study was approved by the local ethics committee, Adviescommissie Wetenschappelijk Onderzoek-Medisch-Ethische Commissie (ACWO-MEC; OLVG Hospital, Amsterdam, ID number 22112, October 2021). The performed interventions are considered part of routine care for which Dutch legislation does not require written informed consent [19].

## Method

The reporting was guided by the Thabane checklist for pilot studies, which was adapted from the CONSORT format for reporting of pilot and feasibility studies that are not randomised [20].

### Study design and setting

In this mixed-methods study, two pharmacy-led approaches were tested between February 2022 and June 2023 in two pragmatically chosen community pharmacies in Amsterdam. The first approach was aimed at reversing prescribing cascades and the second approach at preventing them among patients of 18 years and older. Both approaches were tested in both pharmacies and the pharmacy staff involved were not part of the research team. Ten prescribing cascades were selected from an overview of previously identified problematic prescribing cascades in Dutch primary care for the first approach (Table 1) [6]. For the second approach, five were selected on the following criteria: (1) ≥ 1000 patients in the Netherlands used the index medication in 2022 [21], ensuring it was likely captured during the study period; (2) the ADR typically occurred within a month after dispensing [22, 23], aiding identification; and (3) the ADR was self-detectable (e.g., symptoms, not abnormal laboratory results).

**Table 1:** Prescribing cascades selected for the approach to reverse (number 1–10) and the approach to prevent prescribing cascades (number 6–10)

	Index medication	Adverse drug reaction	Marker medication
1	Proton pump inhibitors	Clostridium difficile infection	Intestinal antiinfectives
2	Lithium	Tremor	Propranolol
3	Lithium	Parkinsonism	Tertiary amines/ Dopaminergics
4	Amiodarone	Hypothyroidism	Thyroid hormones
5	Lithium	Hypothyroidism	Thyroid hormones
6^a^	Dihydropyridines	Peripheral oedema	Loop diuretics
7^a^	ACE-inhibitors	Urinary tract infections	Antibacterials for systemic use
8^a^	ACE-inhibitors	Cough	Cough and cold preparations
9^a^	ACE-inhibitors	Cough	Antihistamines for systemic use
10^a^	Cardiovascular medication^b^	Erectile dysfunction	Phosphodiesterase-5 inhibitors

*ACE* angiotensin converting enzyme

^a^Numbers 6–10 are the index medications that were selected for the approach to prevent prescribing cascades but during the training it was decided to include number 10 only for the ACE-inhibitors and dihydropyridines when addressing numbers 6–9

^b^This includes ACE-inhibitors, loop diuretics, dihydropyridines, statins and beta-blockers potentially causing erectile dysfunction

### Routine care

In Dutch community pharmacies, qualified pharmacy staff members (i.e., pharmacists and technicians) conduct patient counselling on medication use and potential ADRs during the first dispensing. At the second dispensing, patients are typically asked about their medication experiences and any possible ADRs [24, 25]. However, ADRs are not consistently discussed during the second dispensing [26]. Additionally, some patients have their medication home-delivered or do not collect it from the pharmacy themselves, limiting opportunities to discuss ADRs. Furthermore, routine pharmacy care includes efforts to reduce potentially inappropriate prescribing, e.g., through medication reviews aimed at ensuring safe and effective medication use. Although pharmacists use their pharmacy administration and information system (PAIS) to identify patients that may be exposed to suboptimal treatment, there are no explicit strategies or tools available for detecting ADRs that can result in prescribing cascades [24].

### Education and resources

Pharmacy staff received education on general issues related to prescribing cascades, and information on the study aims and procedures to reverse and prevent prescribing cascades. They received a summary card to remind them of the selected prescribing cascades. For both approaches, a step-wise procedure was developed (Table 2). Although the initial screening steps could be conducted by any pharmacy staff member, they were conducted by a pharmacist-researcher (AM) since both pharmacies faced staff shortages that limited their ability to complete all steps within the timeframe of this study.

**Table 2:** Steps for the approach to reverse and the approach to prevent prescribing cascades

Steps	Reversal	Prevention	Performed by^a^
1a	Selection of all patients using the index and marker medication from the PAIS	Selection of all patients being initiated on the index medication from the PAIS to be contacted by phone	Researcher supervised by pharmacist
1b	–	Provide ADR information at first dispensing (routine care)	Pharmacy staff member
2a	Identifying potential prescribing cascades using the PAIS	–	Researcher supervised by pharmacist
2b	–	Phone conversation aimed at identifying ADRs	Researcher supervised by pharmacist
3a	Assessment of potential prescribing cascade without consulting the prescriber		Pharmacist
3b	Assessment together with prescriber to discuss potential medication interventions		Pharmacist
4	If necessary, patient consultation to adjust medication (e.g., lowering dosage, switching medication)		Pharmacist

*ADRs* adverse drug reactions, *PAIS* pharmacy administration and information system

^a^The researcher was a pharmacist not involved in steps 3 and 4. In steps 3 and 4, the community pharmacists evaluated each case to determine whether or not to contact the prescriber and/or propose a change in medication to reverse or prevent a potential prescribing cascade

### Reversing prescribing cascades

For the approach to reverse prescribing cascades, dispensing data from the PAIS was used to identify patients who had been dispensed both index and marker medications between January 2019 and December 2022. A previously developed method was applied to the dispensing data to identify potential prescribing cascades (detailed below) [27]. The community pharmacist evaluated each case to decide whether it was likely a prescribing cascade requiring intervention. If so, the community pharmacist contacted the prescriber to discuss potential changes in treatment.

To identify potential prescribing cascades, a pharmacist-researcher (AM) applied several criteria using dispensing start and end dates. First, the marker should start after the index medication, with no prior use for at least 12-months, ensuring both were the patient’s first dispensings. To increase the likelihood that it concerned a prescribing cascade, the minimum time between the first index and first marker medication was seven days and the maximum time was 12 months [27, 28]. Additionally, the gap between the index medication’s expected end date and the marker’s start date had to be ≤ 4 months, indicating simultaneous use and accounting any potential stock of the index medication. Finally, both index and marker medication had to be in use at data extraction to ensure current potential prescribing cascades.

### Preventing prescribing cascades

For the approach to prevent prescribing cascades, patients were included when there was a first dispensing of an index medication listed in Table 1 between February 2022 and January 2023. Nursing home patients and those without medication history (e.g., new patients) were excluded. Pharmacy staff informed patients about possible ADRs at this first dispensing as part of routine care. One month after this dispensing, the pharmacist-researcher (AM) phoned the patients to ask about drug-related problems. Patients were excluded when they were unreachable by phone after three attempts on different days. Initial phone questions included: ‘*What do you think of the medication so far?’* and ‘*Have you experienced any issues since starting this medication, such as side effects or other effects of the medication?*’ Specific ADR-related questions followed, e.g., ‘*Have you experienced dry cough?* (for ACE-inhibitors). A final general question was asked: ‘*Do you have any questions, concerns, or other problems with your medication?*’ These questions align with the Dutch pharmacy guideline's advice for second dispensing counselling [24]. If a patient experienced an ADR, the pharmacist assessed whether any action was required. In such cases, the pharmacist contacted the prescriber to discuss a possible treatment change.

During training, pharmacy staff expressed concerns about discussing erectile dysfunction related to cardiovascular medications, due to its sensitivity. It was decided to address this ADR only by phone for patients receiving ACE-inhibitors or dihydropyridines.

### Outcome measures

The primary outcome was the percentage of patients for whom a treatment change was initiated to either (a) reverse a prescribing cascade among all patients identified with a potential prescribing cascade, or (b) prevent a potential prescribing cascade among all patients that were contacted by phone. The secondary outcomes included the time investment, potential cost-savings, and the experiences of pharmacists with implementing both approaches.

### Data-collection and classification

Dispensing data and consultation notes were collected from the PAIS by a pharmacist-researcher (AM). For the approach to reverse prescribing cascades, the medication name and start and end dates for each prescription of the index and marker medications were extracted. For the approach to prevent prescribing cascades, these data were extracted for the index medications. Potential ADRs reported by patients were documented and categorised by the researchers as ‘related to a potential prescribing cascades’ and ‘unrelated symptoms’.

For both approaches, the researchers utilised consultations notes and the community-pharmacist’s input to categorise the pharmacists' initial assessments as ‘no intervention needed’, ‘monitoring the patient’, or ‘further assessment with prescriber’. If the prescriber was consulted, the outcomes were further classified as ‘no intervention needed’, ‘monitoring the patient’, ‘intentional prescribing cascade’ or ‘perform intervention’.

Time investment for all steps (Table 2) was estimated by the person conducting the task, either the pharmacist-researcher (AM) or the community pharmacist. Phone conversations were timed, and for steps 2–4, the average time per patient was estimated. Costs were calculated based on the time required for each step, multiplied by Dutch wages for relevant staff in routine practice. Potential savings included reduction in medication costs, dispensing fees, and healthcare visits, assuming cascades would otherwise persist for one year.

Qualitative data included pharmacist feedback and field notes documenting problems. An online meeting with both pharmacists provided further insights for implementation of the tested approaches, summarized in a report and reviewed by the pharmacists for accuracy.

### Data analysis

For this proof-of-concept study no formal sample size calculation was conducted. All data were collected and analysed in Excel 2016 (Microsoft Corporation, Redmond, WA, USA) using descriptive statistics.

## Results

### Reversing prescribing cascades

**Fig. 1:**
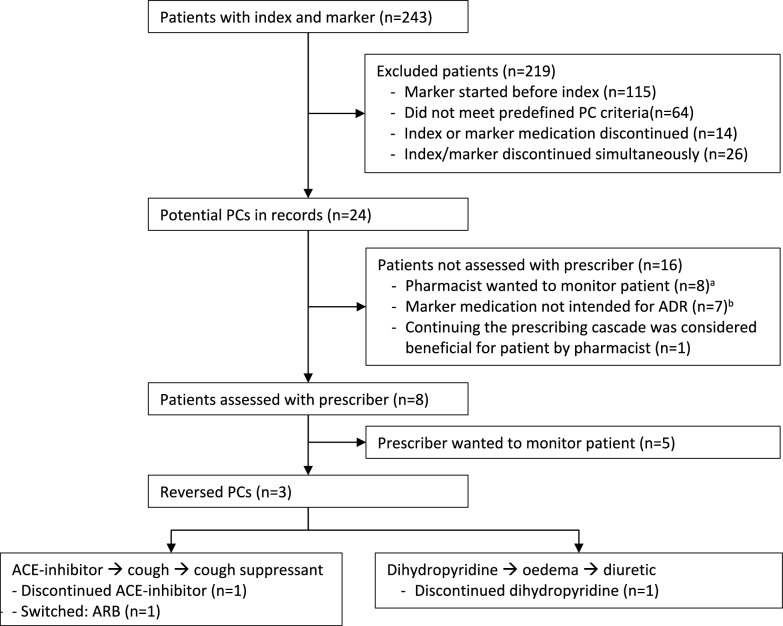
Patient flow of the approach to reverse prescribing cascades. ^a^For example, when a cough suppressant or antibacterial was dispensed only once and the pharmacist decided to monitor the patient to wait for repeated prescriptions of the marker medication. ^b^For example, an antibacterial agent used for dental procedures. *PC* prescribing cascade, *ADR* adverse drug reaction, *ACE* angiotensin converting enzyme, *ARB* angiotensin II receptor blocker

During the study period, 243 patients received both an index and marker medication (Fig. 1). Of these, 115 used the marker before the index medication, and 64 did not meet the predefined criteria for prescribing cascades (n = 64). Fourteen patients had either the index or marker medication discontinued (n = 14), and 26 had both discontinued. The reasons for discontinuation were unknown.

The remaining 24 patients with potential prescribing cascades were included, 16 of whom were assessed by the community pharmacist without contacting the prescriber. For eight patients, the pharmacist preferred to monitor the patient, including those on ACE inhibitors causing cough (treated with cough suppressants) and ACE inhibitors causing urinary tract infections (treated with antibiotics). In seven cases, the marker medication was unrelated to an ADR (e.g., antibiotics for dental procedures). For one patient, continuing the diuretic was deemed beneficial as it treated oedema from kidney disease, not due to dihydropyridine.

The remaining eight patients were assessed with the prescriber, with interventions to reverse prescribing cascades for three patients (12.5% of 24 patients). One prescribing cascade –dihydropyridine causing oedema– was reversed by switching to an ACE-inhibitor. Two other prescribing cascades –ACE-inhibitor causing cough– were reversed by switching to an angiotensin-II-receptor blocker and discontinuing the ACE-inhibitor. These three prescribing cascades had persisted between three months and two years prior to intervention.

For the remaining five patients, the prescriber wanted to monitor the patient. There were no further follow-up data collected.

### Preventing prescribing cascades

**Fig. 2:**
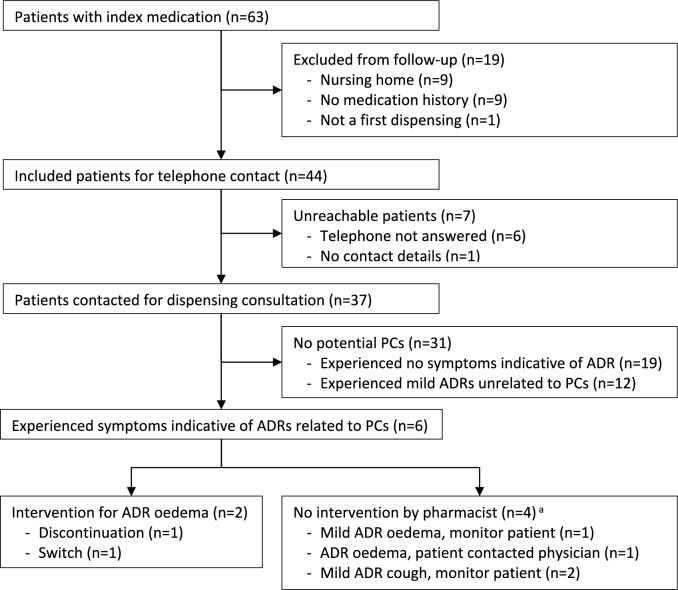
Patient flow of the approach to prevent prescribing cascades. ^a^Monitoring was not considered as an intervention. *PC* prescribing cascade, *ADR* adverse drug reaction

Among 63 patients receiving a first dispensing of an ACE-inhibitor or dihydropyridine (Fig. 2), 19 were excluded, mainly due to nursing home residency (n = 9) or lack of a medication history (n = 9). Of the remaining 44 patients, seven were unreachable. Among the 37 contacted patients, 19 reported no ADRs and 12 had mild unrelated symptoms not requiring an intervention.

Six patients reported symptoms linked to potential prescribing cascades: two had cough with ACE-inhibitors, and four had oedema with dihydropyridines. One patient had already contacted the prescriber, leading to dihydropyridine discontinuation. Three patients experienced mild symptoms, requiring no intervention. For the other two patients (4.5% of 44 patients) changes in treatment were initiated after contacting the prescriber who agreed with the proposed changes. One discontinued amlodipine due to the patient's adequate blood pressure, the other switched from amlodipine to barnidipine following the cardiologist's advice, then to losartan with spironolactone when oedema persisted.

### Time investment and cost-savings

For the approach to reverse prescribing cascades, the time investment for selecting and identifying 24 patients with a potential prescribing cascade was 270 min (4.5 h). The pharmacist’s assessment of these 24 patients, including consultations, required an additional 110 min (1.8 h) (Table 3).

**Table 3:** The estimated time investment to reverse or prevent prescribing cascades

	Reverse	Prevent
	Time in minutes (n = number of patients)	Time in minutes (n = number of patients)
1a.Selection of patients on index and marker respectively initiation index medication from the PAIS	60	50
2a.Identifying potential prescribing cascades using the PAIS	210 (n = 24)	n.a
2b.Identifying potential ADRs by contacting patients	n.a.	228 (n = 37) 12 (n = 24 times not reached)^a^
3a.Assessment of patients by the pharmacist without consulting the prescriber	32 (n = 16)	9 (n = 4)
3b.Assessment by both the pharmacist and prescriber to discuss potential medication interventions	48 (n = 8)	11 (n = 2)
4.Patient consultation to conduct a medication intervention	30 (n = 3)	10 (n = 2)
Time investment steps 1 and 2	270	290
Time investment steps 3 and 4	110	30
Total	380 (6.3 h)	320 (5.3 h)

*n.a.* not applicable, *PAIS* pharmacy administration and information system

^a^This total included calls not answered by patients that were reached on the second or third attempt

For the approach to prevent prescribing cascades, the time investment for selecting and trying to contact 44 patients potentially experiencing an ADR was 290 min (4.8 h). The pharmacist’s assessment of six patients, including consultations, required 30 min (0.5 h) (Table 3).

The overall time investment can be cost-saving when labour costs of the intervention are compared with the costs of potentially unnecessary medication, dispensing fees and general practitioner visits, assuming the prescribing cascade would last one year without the intervention. The estimated labour costs for reversing cascades were around €213 and the savings around €445 (Supplement 1). The estimated labour costs for preventing cascades were €168 and the savings €178.

### Experiences with implementing the approaches

For the approach to reverse prescribing cascades, both pharmacists mentioned that a single dispensing of the marker medication (e.g., antibiotics) may be insufficient to warrant an intervention for some prescribing cascades. They preferred focusing on patients with repeated dispensings of the marker medication. Furthermore, a lack of information on the indication of medications limited their assessment, requiring prescriber consultation for relevant details. In addition, limited knowledge of appropriate interventions for prescribing cascades was also mentioned. Both pharmacists reported that patients responded well to the service. The pharmacists viewed both approaches as valuable. They felt the study raised awareness about lesser-known prescribing cascades and the importance of addressing ADRs. To reduce the time investment, algorithms were suggested as screening tools for potential prescribing cascades. Supplement 2 provides a summary of the pharmacists’ experiences.

## Discussion

This is the first study testing pharmacy-led interventions to reverse or prevent prescribing cascades. Of 24 patients with potential prescribing cascades identified from dispensing records, three (12.5%) received interventions to reverse a cascade. Of 63 patients who were initiated on medication included in the approach to prevent prescribing cascades, 44 were contacted by phone, with two (4.5%) receiving interventions. Both approaches were time-intensive, requiring extensive screening to identify actionable cases. While pharmacists viewed both approaches positively, they expressed the need for automated screening strategies and additional support for managing prescribing cascades.

A strength of this study is testing both approaches in two community pharmacies, collecting quantitative and qualitative outcomes. Nonetheless, the small sample size limits generalisability, and larger studies are needed. Another limitation is the delegation of the screening steps to the pharmacist-researcher, which may have introduced bias and also limits the generalisability. Future studies should assess the feasibility of pharmacy staff conducting these steps. Since it is not likely that pharmacy staff would need less time to conduct these steps, automated screening procedures are clearly needed. Time investment for most steps was estimated, reducing precision. Additionally, a one year period was arbitrarily chosen for calculating intervention costs and savings due to limited data on the typical duration of prescribing cascades. Also, costs from the patient perspective were not included.

Healthcare providers may face challenges in reversing or preventing prescribing cascades, including lack of awareness and hesitation to intervene [18, 29]. Although this study raised awareness, pharmacists and prescribers often opted to monitor patients instead of acting. This hesitation may stem from insufficient information on the marker medication's indication, as noted previously [2]. Notably, the selected prescribing cascades involved well-known ADRs, such as cough from ACE-inhibitors and oedema from dihydropyridines, but interventions were withheld for mild symptoms. The short follow-up period of this study did not allow for evaluating whether symptoms persisted and later required intervention. Healthcare providers’ reluctance to act when patients were stable has been previously identified [2], which may be particularly the case in the complex context of polypharmacy [29].

Qualitative research making use of theoretical frameworks helps to understand what is needed for addressing prescribing cascades [30]. Educating both healthcare providers and patients is key to reversing or preventing prescribing cascades [30, 31]. The pharmacists in this study noted the lack of specific guidance on the identification and management of prescribing cascades. While the pharmacy staff received general education on the topic, no explicit recommendations were provided on interventions. A qualitative study concluded that educational materials on prescribing cascades for Alzheimer’s patients can help healthcare providers [32]. Another study highlighted the need for tools to detect prescribing cascades, as noted by the pharmacists in this study [31]. Finally, although there is a Dutch guideline to support pharmacy staff in patient consultation, they may need more training in consultation skills related to addressing ADRs [24].

No studies have estimated the proportion of prescribing cascades currently reversed in clinical practice. In the approach to reverse prescribing cascades, 26 patients discontinued both index and marker medications simultaneously, some of which may have been cascades. Early ADR detection is essential to prevent cascade initiation and progression. One prescribing cascade identified in this study had lasted for two years. Prompt intervention can significantly reduce prolonged exposure to unnecessary medications and associated risks [33].

Future efforts are needed to develop efficient patient screening methods. Algorithms similar to those used for inappropriate prescribing should be developed to identify cascades [34]. Implementing such tools within pharmacy-led interventions appears feasible [16]. Also, electronic tools or (online) patient questionnaires could enhance timely ADR identification [31].

Awareness of prescribing cascades needs to increase among healthcare providers and patients. Pharmacists' concerns about knowledge gaps highlight the need for improved training and education, as current programs largely overlook this topic [35]. Medication handbooks offer limited guidance, though a section on prescribing cascades was recently added to the Dutch Pharmacotherapy Compass [36].

## Conclusion

This study showed that pharmacy-led approaches can lead to the reversal and prevention of prescribing cascades but more efficient screening methods and tools are needed before a broader implementation can be recommended.

## Supplementary Information

Below is the link to the electronic supplementary material.

Supplementary file1 (DOCX 36 KB)
