# Supplementary material for: Pharmacy-led interventions to reverse and prevent prescribing cascades in primary care: a proof-of-concept study
Source: Int J Clin Pharm. 2025 Feb 15;47(3):784–93. doi: 10.1007/s11096-025-01873-8 (PMC12125053; doi:10.1007/s11096-025-01873-8)
Supplement: Supplementary file 1 — Supplementary file1 (DOCX 36 KB) [file 11096_2025_1873_MOESM1_ESM.docx]

**Supplementary materials**

**Title: Pharmacy-led interventions to reverse and prevent prescribing cascades in primary care: a proof of concept study**

**Running head: Reversing and preventing prescribing cascades**

Atiya K Mohammad^a,b^, Jacqueline G Hugtenburg^c^, Yildiz Ceylan^d^, Marcel Kooij^e^, Saskia Knies^f^, Patricia MLA van den Bemt^b^, Petra Denig^b^, #Fatma Karapinar-Çarkıt^a,g,h^.

^a^ Department of Clinical Pharmacy, OLVG Hospital, Amsterdam, The Netherlands

^b^ Department of Clinical Pharmacy and Pharmacology, University Medical Centre Groningen, Groningen, The Netherlands

^c^ Department of Clinical Pharmacology and Pharmacy, Amsterdam UMC, location VUMC, Amsterdam, The Netherlands

^d^ Community pharmacy, Benu Apotheek Wester, Amsterdam, The Netherlands.

^e^ Community pharmacy, Service Apotheek Koning, Amsterdam, The Netherlands.

^f^ The National Health Care Institute (Zorginstituut Nederland), Diemen, The Netherlands

^g^ Department of Clinical Pharmacy & Toxicology, Maastricht University Medical Center+, Maastricht, Netherlands

^h^ Department of Clinical Pharmacy, CARIM, Cardiovascular Research Institute Maastricht, Maastricht University, Maastricht, Netherlands

**#Corresponding author:**

Fatma Karapinar-Çarkıt, PharmD, PhD

Maastricht University Medical Center+, Department of Clinical Pharmacy & Toxicology

P. Debyelaan 25, 6229 HX, Maastricht, the Netherlands,

Tel: +31(0)43-3871881

e-mail: [f.karapinar@mumc.nl](mailto:f.karapinar@mumc.nl)

**Supplement 1 - Cost-savings analysis**

**Cost-savings analysis**

The cost-savings analysis was performed by comparing the labour costs of healthcare providers against the savings from reversing or preventing a prescribing cascade.

**Labour costs**

For the labour costs, the time needed to perform the following tasks was considered: 1) patient selection, 2) identification of potential prescribing cascades (reversal) or identification of ADRs through contacting patients (prevention of prescribing cascades), 3) assessment by pharmacists to determine whether an intervention was needed, 4) discussion with prescribers about a possible intervention, and 5) if necessary, advice to patients. This time was then multiplied by the salary of the performing pharmacy staff.

Salary

To calculate the salary of pharmacy staff, the yearly workable hours were first calculated [1]. For a general 40-hour workweek, there were 1701.8 workable hours per year (Table 1). This workweek was used for all pharmacy staff in this study.

**Table 1** Workable hours per year for a 40-hour workweek

|  | Calculation | Number of hours/year |
| --- | --- | --- |
| Basic hours (40-hour workweek) | 40 hours x 52 weeks | 2080 |
| Public holidays | (average) 6 days x 8 hours | 48 |
| Vacation/annual leave | 9% of basic hours | 187.2 |
| Sick leave | 5% of basic hours | 104 |
| Special leave | 1 day | 8 |
| Study and training | 2 days | 16 |
| Extra age-dependent hours^a^ | 15 hours | 15 |
| Workable hours (basic hours minus non-workable hours) |  | 1701.8 |

^a^ In the Netherlands, older pharmacy staff can have more free hours.

The monthly and hourly salary of each employee were calculated according to the following: For a community pharmacist, the monthly salary was €4,224.14 (6th year of working experience [2]). The hourly salary costs were calculated as follows: (€4,224.14 x 12) / 1701.8 = €29.79. These costs were then increased by the employer costs (e.g., insurance, pension): €29.79 + (€29.79 x 39%) = €41.40.

**Table 2** The monthly and hourly salary per employee

|  | Monthly salary^a^ | Hourly salary costs |
| --- | --- | --- |
| Community pharmacist | €4,224.14 | €41.40 |
| Pharmaceutical consultant^b^ | €3,107.21 | €30.45 |

^a^ 6^th^ year of working experience [2].

^b^ In the Netherlands, a pharmacy technician can specialize further into pharmaceutical consultant, following a 3-year training focused on pharmacotherapy and communication [3, 4].

**Savings**

For the potential savings, prices for the first dispensing of medication, the medication prices per prescribing cascade, and standard dispensing were included. A visit to the general practitioner was added for the preventing approach (Table 3).

**Table 3** The prices for each saving

| Savings | | Price |
| --- | --- | --- |
| First dispensing | | €13.50 |
| Standard (second) dispensing | | €6.50 |
| General practitioner visit | | €30.87 |
| Medication per tablet | Furosemide 40 mg^a^ | €0.05 |
|  | Codeine 20 mg^b^ | €0.41 |

^a^ Furosemide was given as treatment for dihydropyridine potentially causing oedema treated with diuretics.

^b^ Codeine was given as treatment for angiotensin converting enzyme-inhibitors potentially causing cough treated with cough suppressants.

In the Netherlands, the first dispensing of medication is for fifteen days. Future dispensings are generally for 90 days. For the savings it is assumed that the prescribing cascade would continue for one year in the event that no pharmacy-led intervention was performed.

**Cost-saving to reverse prescribing cascades**

Labour costs

For the labour costs we assume that the steps performed by the researcher are performed by a pharmaceutical consultant in practice. Pharmaceutical consultants are pharmacy technicians with three year additional training compared to pharmacy technicians

|  | Time investment (hours) | Hourly wage | Labour costs |
| --- | --- | --- | --- |
| Pharmacist | 1.83 | €41.40 | €75.76 |
| Pharmaceutical consultant | 4.50 | €30.45 | €137.05 |

Savings

In total, there were three patients for whom a prescribing cascade was reversed. For one patient, it concerned dihydropyridine potentially causing oedema treated with diuretics, and for two patients, it concerned ACE inhibitors potentially causing cough treated with cough suppressants.

*Savings to reverse prescribing cascades*

The savings were calculated with the assumption that there was an initial dispensing for 15 days, followed by four subsequent standard dispensings of 90 days each.

Savings = €13.50 first dispensing + (15 days x € medication price per day) + (4 x (€6.50 standard dispensing + (90 days x € medication price per day)))

Example calculation for the prescribing cascade of dihydropyridine potentially causing oedema treated with diuretics:

Savings = €13.50 + (15 x €0.05) + (4 x (€6.50 + (90 x €0.05))) = €58.25 per year.

For ACE inhibitors potentially causing cough treated with cough suppressants, the savings were €193.25 per year.

Cost-saving difference

| Total labour cost | Saving per prescribing cascade | | Cost saving |
| --- | --- | --- | --- |
|  | *Dihydropyridines* | *ACE-inhibitor* |  |
| €212.81 | €58.25 | €193.25^a^ | -€231.96 |

^a^ Multiplied by two, as two interventions were performed for this prescribing cascade.

**Cost-saving to prevent prescribing cascades**

Labour costs

|  | Time investment (hours) | Hourly wage | Labour costs |
| --- | --- | --- | --- |
| Pharmacist | 0.5 | €41.40 | €20.70 |
| Pharmaceutical consultant | 4.83 | €30.45 | €147.20 |

Savings

In total, there were two patients for whom a prescribing cascade was prevented. For both patients, it concerned the prescribing cascade of dihydropyridine potentially causing oedema treated with diuretics. The pharmacist contacted the general practitioner by phone for both patients. Without this intervention, the patients could have visited the general practitioner for the ADR. Therefore, the prevention of one general practitioner visit was added.

*Savings to prevent prescribing cascades*

The savings were calculated with the assumption that one general practitioner visit would be needed, an initial dispensing for 15 days, followed by four subsequent standard dispensings of 90 days each.

Yearly saving costs = €30.87 general practitioner visit + €13.50 first dispensing + (15 days x € medication price per day) + (4 x (€6.50 standard dispensing + (90 days x €medication price per day))).

For the prescribing cascade of dihydropyridine potentially causing oedema treated with diuretics, the savings would have been €89.12 per year.

Cost-saving difference

| Total cost labour | Saving per prescribing cascade | | Cost saving |
| --- | --- | --- | --- |
|  | *Dihydropyridines* | *ACE-inhibitor* |  |
| €167.90 | €89.12 ^a^ | Not applicable | -€10.34 |

^a^ Multiplied by two, as two interventions were performed for this prescribing cascade.

**Supplement 2 – Pharmacists’ experiences with approaches**

**Pharmacist A**

This study has raised awareness among doctors and pharmacists, especially regarding less well-known prescribing cascades. Training our pharmacy staff has led to a better understanding of the importance of explicitly asking patients about their medication experience and the occurrence of adverse drug reactions (ADRs).

A patient who moved to another region and therefore changed his pharmacy phoned us to explicitly thank us for the consultation in this study and for the good care regarding ADRs.

**Pharmacist B**

This study has resulted in increased attentiveness among pharmacy technicians regarding potential ADRs after the training and more attention was given to it during the dispensings in the pharmacy. Not all staff members were familiar with these ADRs and prescribing cascades. This study has certainly contributed to an increased awareness of these issues. Patients responded positively to questions about ADRs.

**Feedback from both pharmacists for adaptations**

In order to implement this approach in practice, clear guidelines are needed on how to address prescribing cascades. Situations were encountered where it was unclear whether interventions should be performed or which interventions were appropriate. Regarding the approach to reverse prescribing cascades, general practitioners sometimes experienced difficulties in assessing whether these were actually prescribing cascades. This occurred because the considerations of the initial prescriber or the indication for the marker medication were unknown. Prescribers were also hesitant to act because the patient was stable, and disrupting a stable patient was considered undesirable.

For future research, interventions are needed with both a reactive and a proactive part. The reactive part should incorporate prescribing cascades into tools in such a way that a notification is received in the pharmacy information system when a marker medication is prescribed and a patient has been using the associated index medication. A drawback of this approach is that a patient already experienced an ADR and possibly visited a physician, which you would rather avoid. Therefore, the reactive part of the intervention should be augmented with a proactive part. An app could be developed where patients can answer a short questionnaire about ADRs when they want to order a repeat prescription for chronic medication. This can increase the efficiency of identifying ADRs requiring attention of the pharmacist. For other patients, a pharmaceutical consultant could conduct second dispensing consultations for multiple pharmacies (rather than each pharmacy assigning a staff member separately) and coordinate with the patient's pharmacist where necessary.

Currently, asking all patients about ADRs to prevent prescribing cascades is not possible because 1) it takes a lot of time and there is a shortage of staff members in most pharmacies, and 2) not all patients come to the pharmacy to pick up their chronic medication. Most chronic medication is being delivered to patients at home or picked up by caregivers.

**References**

1. Hakkaart-van Roijen L PS, Kanters T. Cost guide for economic evaluations in healthcare: Methodology and reference prices (in Dutch). 2024.

2. The Royal Dutch Association of Pharmacists (KNMP). Collective Labor Agreement for Pharmacists in employment (in Dutch); 2022. Available from: <https://www.knmp.nl/sites/default/files/2022-09/Cao%20apothekers%20in%20dienstverband%202022-2023.pdf>. Accessed 04 Sept 2024.

3. Daliri S, Hugtenburg JG, Ter Riet G, et al. The effect of a pharmacy-led transitional care program on medication-related problems post-discharge: A before-After prospective study. PLoS One. 2019;14(3):e0213593.

4. Karapinar-Carkit F, Borgsteede SD, Zoer J, et al. The effect of the COACH program (Continuity Of Appropriate pharmacotherapy, patient Counselling and information transfer in Healthcare) on readmission rates in a multicultural population of internal medicine patients. BMC Health Serv Res. 2010;10:39.
